# Partial Thickness Rotator Cuff Tears: Current Concepts

**DOI:** 10.1155/2015/458786

**Published:** 2015-06-11

**Authors:** Graeme Matthewson, Cara J. Beach, Atiba A. Nelson, Jarret M. Woodmass, Yohei Ono, Richard S. Boorman, Ian K. Y. Lo, Gail M. Thornton

**Affiliations:** ^1^Department of Surgery, Section of Orthopaedic Surgery, McCaig Institute for Bone and Joint Health, University of Calgary, Calgary, AB, Canada T2N 4Z6; ^2^Department of Orthopaedic Surgery, Nagoya University Graduate School of Medicine, Nagoya, Aichi 466-8550, Japan; ^3^Department of Orthopaedics, University of British Columbia, Vancouver, BC, Canada V5Z 1M9

## Abstract

Partial thickness rotator cuff tears are a common cause of pain in the adult shoulder. Despite their high prevalence, the diagnosis and treatment of partial thickness rotator cuff tears remains controversial. While recent studies have helped to elucidate the anatomy and natural history of disease progression, the optimal treatment, both nonoperative and operative, is unclear. Although the advent of arthroscopy has improved the accuracy of the diagnosis of partial thickness rotator cuff tears, the number of surgical techniques used to repair these tears has also increased. While multiple repair techniques have been described, there is currently no significant clinical evidence supporting more complex surgical techniques over standard rotator cuff repair. Further research is required to determine the clinical indications for surgical and nonsurgical management, when formal rotator cuff repair is specifically indicated and when biologic adjunctive therapy may be utilized.

## 1. Introduction

Partial thickness rotator cuff tears (PTRCTs) are a common pathology that may significantly impact a spectrum of patients including sedentary individuals, workers, and athletes. Despite their high prevalence, the majority of studies on the treatment of rotator cuff tears have focused on full thickness tears. PTRCTs have been relatively ignored and subsequently the treatment of PTRCTs remains controversial. Currently, most studies on the treatment of PTRCTs have described surgical techniques or outcomes; few studies have focused on the etiology or the conservative management of PTRCTs. This has led to a poor understanding of the natural history of the disease process and has compounded the debate over their optimal treatment. The purpose of this review was to evaluate the current state of knowledge regarding PTRCTs including prevalence, etiology, diagnosis, and treatment.

## 2. Anatomy

The classical description of the rotator cuff involves a convergence of 4 tendons: supraspinatus, infraspinatus, teres minor, and subscapularis. These tendons form a multiple layered horseshoe shape flattened architecture which inserts onto the humeral head [1]. When viewed from the glenohumeral joint, the superior insertion of the rotator cuff generally appears as a thickening of the capsule (the rotator cable) surrounding a thinner area of tissue (the crescent region), which inserts into the greater tuberosity. The rotator cable extends from the biceps anteriorly to the inferior margin of the infraspinatus tendon posteriorly. This thickening, as described by Burkhart et al. [2], is thought to mechanically protect the weaker avascular crescent region where partial thickness rotator cuff tears most commonly occur.

**Figure 1:**
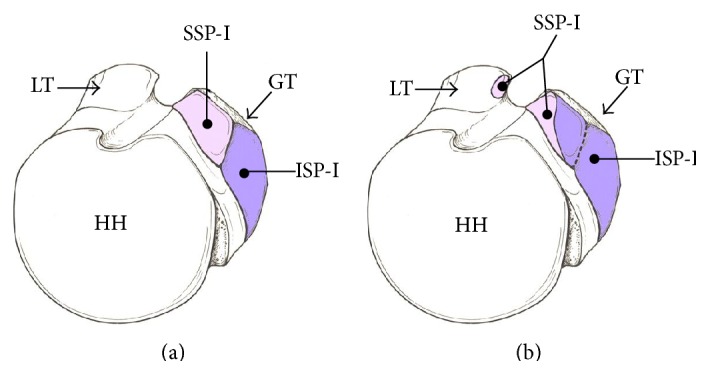
Illustration showing the two different insertional descriptions of the humeral insertions of the supraspinatus (SSP-I) and infraspinatus (ISP-I). (a) The generally accepted concept of the supraspinatus inserting into the highest impression and the infraspinatus inserting into the middle impression of the greater tuberosity (GT). (b) An alternative illustration, in which the insertion of the infraspinatus occupies the majority of the GT, covering both the middle and half of the highest impression of the GT. Also noted is the additional attachment of the supraspinatus to the most superior aspect of the lesser tuberosity (LT). HH = humeral head (adapted and reprinted from [6] with permission).

In 1992, Clark and Harryman [1] demonstrated that the rotator cuff insertion contained 5 distinct histologic layers. The 1st layer comprised the superficial coracohumeral ligament. The 2nd and 3rd layers contain the tendinous fibres of the rotator cuff. The 4th and 5th layers consist of the arterioles and loose connective tissue adjacent to the bone. Although this horseshoe shaped insertion may have interdigitations between the rotator cuff tendons, Curtis et al. [3] demonstrated that there were indeed separate footprints of each tendon, with a wide range of widths and lengths (Table 1). Curtis and others [3–5] described a relatively classic straight medial-to-lateral directional insertion of the rotator cuff tendon to bone (Figure 1(a)).

Recently, a cadaveric study [6] offered an alternative description of the supraspinatus and infraspinatus insertions into the greater tuberosity. Mochizuki et al. [6] described a more curvilinear insertion of the infraspinatus tendon wrapping anteriorly around the superior aspect of the greater tuberosity. They demonstrated that the infraspinatus tendon consumed a large portion of the lateral aspect of the superior facet of the greater tuberosity, an area generally considered part of the supraspinatus tendon insertion. In contrast to classical descriptions of the rotator cuff as described above [3], the supraspinatus tendon inserted on only a small portion of the most anterior aspect of the greater tuberosity and, in 21% of cases, fibers had inserted into the lesser tuberosity [6] (Figure 1(b)).

However, while the precise anatomy of the rotator cuff insertion continues to evolve, most classification systems and clinical algorithms of how to treat PTRCTs rely on the classic descriptions as described by Clark and Harryman, Curtis et al., and others [1, 3–6] (Figure 1(a)).

## 3. Prevalence

Based on cadaveric and imaging studies [25–28], the prevalence of PTRCTs ranges from 13% to 32%, in part, related to its strong correlation to patient age. In one MRI study of asymptomatic individuals [28], the overall prevalence of PTRCTs was 20%. In patients under the age of 40, the prevalence was approximately 4%; whereas, in patients over the age of 60, the prevalence was 26%. This age-related difference in prevalence was supported by Milgrom et al. [29] who found a prevalence of full or partial thickness rotator cuff tears of 5%–11% in subjects aged 40–60 but 80% in those aged 70 years or older. They demonstrated a linear increase in the prevalence of rotator cuff tears after the 5th decade of life.

However, the true prevalence of PTRCTs may in fact be underreported. Investigation of 249 cadaveric supraspinatus tendons revealed that 13% had PTRCTs, of which 55% were intratendinous, 27% were articular surface, and 18% were bursal surface [30, 31], suggesting that the vast majority of these intratendinous tears may have gone unnoticed in prior studies, due to the difficulties in identifying intratendinous tears with diagnostic imaging.

Similarly, the prevalence of PTRCTs is surprisingly high in overhead athletes. In 2003, Connor et al. [32] performed MRIs in the shoulders of asymptomatic elite overhead athletes. In twenty athletes, the overall prevalence of rotator cuff tears (i.e., partial or full thickness) was 40% in the dominant throwing shoulder. Importantly, at a 5-year follow-up, none of the athletes developed shoulder symptoms requiring treatment, and none of them had appreciable decreases in their level of play.

Collectively, these results highlight the significant underlying prevalence of PTRCTs. Furthermore, while MRI or other modalities can detect the presence of a PTRCT, correlating MRI findings with clinical presentation is critical in determining if the existing pathology is responsible for the patient's symptoms.

## 4. Etiology and Pathogenesis

The etiology and pathogenesis of PTRCTs is likely multifactorial with both intrinsic and extrinsic factors contributing to an individual's rotator cuff lesion. Intrinsic factors, including age-related microscopic changes (hypocellularity, fascicular thinning, and granulation tissue) and decreased vascularity of the tissues, predispose a tendon to degenerative tearing and alterations in intratendinous strain [26, 27, 33–35]. Extrinsic factors, including subacromial impingement, glenohumeral instability, and internal impingement [36–38], can further contribute to anatomic pathology. Finally, traumatic events, either singular in nature or repetitive (e.g., overhead athlete), can eventually contribute to tensile overload and fiber failure of the rotator cuff. While still unclear, the presumption is that because of increased tendon strain due to the presence of a tear, PTRCTs generally increase in size over time [34].

A few studies have evaluated the natural history of PTRCTs. Historically, in 1994, Yamanaka and Matsumoto [39] reported that after a mean follow-up time of 1.1 years 28% of PTRCTs had progressed to full thickness and 80% of the PTRCTs increased in size over this short time period. However, a number of more current studies have suggested that PTRCTs may not progress as rapidly as previously presumed [40–42]. In a more recent study, 37 patients were evaluated by serial MRI or MR arthrography. At a mean of 4.4 years postoperatively, 76% of patients had no significant progression of their PTRCT, 16% had an increase in tear size, and 8% progressed to a full thickness tear [40]. Furthermore, they showed a significant correlation between the risk of tear progression and percentage of the tendon thickness involved at presentation. In patients with tears involving ≥ 50% of the tendon thickness, 55% had tear progression; whereas, in patients with tears involving < 50% of the tendon thickness, only 14% had tear progression.

The majority of imaging studies have demonstrated that healing of PTRCTs is, in fact, rare [39, 41, 43]. This is further supported by histologic studies by Fukuda et al. [44, 45] who demonstrated that PTRCTs did not have the ability to heal themselves over time. Furthermore, it appears that nonanatomic procedures that do not specifically address the PTRCT do not prevent tear progression. In one study by Hyvonen et al. [46], 93 patients were followed for a mean of 9 years following subacromial decompression for impingement syndrome. However, subacromial decompression did not appear to prevent the progression of rotator cuff tearing. Patients who reported excellent results had a 4% tear rate (2 full thickness); good results, a 25% tear rate (4 full thickness and 2 articular surface); fair results, a 33% tear rate (5 full thickness and 1 bursal surface); and poor results a 55% tear rate (1 full thickness and 4 partial thickness, 1 bursal surface and 3 articular surface) on MRI or single contrast arthrography.

Therefore, since PTRCTs are secondary to age-related degenerative change within an altered biomechanical environment, progression of the tear can occur. Furthermore, this can occur even when surgical procedures that do not address the primary pathology are performed.

## 5. Diagnosis

While pain is the most common symptom, the clinical presentation of PTRCTs can vary widely. Common presentations include a painful arc of motion, crepitus, weakness, and positive impingement signs [33]. In addition, difficulties with overhead activities or overhead sports are common [35]. Due to the high prevalence of asymptomatic PTRCTs (as discussed above), the correlation of clinical findings to imaging studies is crucial.

Ultrasonography is a reliable and cost-effective tool in the accurate detection of full thickness rotator cuff tears [47, 48]. However, its utility can be limited in the detection of PTRCTs, due to the difficulties in distinguishing PTRCTs from tendon scarring or small full thickness lesions and its inability to detect glenohumeral pathology. Despite increasing usage, ultrasonography continues to be reliant on the operator's technique and has resulted in a wide variability in results limiting its widespread acceptance [48].

However, in expert hands, Teefey et al. [49] reported similar efficacy for ultrasonography and MRI in detecting PTRCTs as confirmed by arthroscopy (13/19 tears and 12/19 tears, resp.). Furthermore, a recent meta-analysis [50] of 65 studies assessing diagnostic imaging of rotator cuff tears reported similar sensitivity and specificity of ultrasound and MRI at identifying PTRCTs. Sensitivities of 66.7% and 63.6% and specificities of 93.5% and 91.7% were reported, respectively.

While MRI has limits in its ability to accurately detect PTRCTs, MR arthrography remains the imaging modality of choice. Its high mean sensitivity (85.9%) and specificity (96.0%) place it superior to other imaging modalities [50]. MR arthrography is particularly accurate in identifying articular surface rotator cuff tears, and its sensitivity may be further enhanced by imaging in an abducted and externally rotated position [50].

Despite advances in imaging technologies, arthroscopy remains the gold standard for diagnosing PTRCTs [51]. Arthroscopy allows direct visualization of the bursal and articular surfaces of the rotator cuff as well as the anatomic footprint. In addition, arthroscopy provides the ability to probe the soft tissues to identify areas of tearing that would otherwise be undetectable. Several techniques have been used to aid in the intraoperative assessment of PTRCTs including the use of methylene blue (i.e., Fukuda colour test) [44], suture marking (i.e., Snyder suture technique) [52], and the “bubble sign” (i.e., Lo bubble sign) for intratendinous tears [53].

Regardless of the technique used, arthroscopy allows direct visualization of the pathology at hand and the opportunity to directly assess the degree of tearing and the quality of the remaining tissue. These characteristics may be important when determining the optimal surgical treatment (e.g., debridement versus repair) making arthroscopy advantageous when compared to other less invasive diagnostic modalities.

## 6. Classification

PTRCTs can be classified by location (articular, bursal, and intratendinous), the tendons involved (supraspinatus, infraspinatus, teres minor, and subscapularis), and the size of the tear (represented as percentage of the tendon thickness torn). The Ellman classification defines tears based on location (articular, bursal, and intratendinous) and the percentage of the tendon thickness torn (Table 2) [8]. While widely accepted, this classification system does not take into account a number of factors including: an analysis of tissue quality, the area of tearing (i.e., not just thickness but anterior to posterior and medial to lateral), or the etiology of the tear itself. Furthermore, there is relatively poor interobserver reliability [54] of this classification system when using imaging modalities (e.g., MRI) or even dedicated arthroscopic videos [55]. Despite this, the Ellman classification system continues to be the most popular classification system quoted, likely due to its history of utilization and that no alternative classification system has gained universal acceptance.

## 7. Nonoperative Treatment

The optimal treatment of PTRCTs is multifactorial and may be influenced by factors including the patient's age, symptoms, functional deficit, size of the tear, tear location (e.g., bursal versus articular), nature of onset (e.g., degenerative versus traumatic), etiology, and vocation and avocation activities. In the majority of cases, a trial of nonoperative treatment (e.g., activity modification, NSAIDs, pain medications, physiotherapy, and steroid injection) is reasonable since, unlike full thickness rotator cuff tears, the risk of fatty infiltration, muscular atrophy, and significant tear extension are relatively minimal. However, the success of nonoperative treatment of PTRCTs has rarely been reported.

One study [40] reviewed 76 consecutive patients with PTRCTs in which 50% were treated nonoperatively. At a mean of approximately 4 years of follow-up, 91% of patients were still satisfied with nonoperative treatment. Patients who had an atraumatic onset of symptoms involving the nondominant extremity, as well as a tear that involved < 50% of the tendon thickness, were more likely to be treated nonoperatively. While this study evaluated patients in the general population, nonoperative treatment for PTRCTs may actually be preferable in certain athletic populations. In the throwing athlete, due to the time off, stiffness, and decreased range of motion associated with surgery, conservative management is the treatment of choice for tears involving up to 75% of the tendon thickness [56].

Clearly further research is required to determine the effectiveness of nonoperative treatment in the different patient populations (e.g., old versus young) and different tears (e.g., bursal versus articular and traumatic versus atraumatic). While PTRCTs can be treated successfully nonoperatively, clinical success, particularly in the short term, must be balanced against the potential for long-term anatomic disease progression.

## 8. Operative Treatment

Surgical treatment of PTRCTs is generally indicated in patients with failure of nonoperative treatment for 3–6 months and in younger patients with a traumatic injury. While a number of surgical options exist (i.e., debridement, decompression, and repair), the major surgical decision required is whether the patient may benefit from rotator cuff debridement +/− subacromial decompression or if a formal repair of the PTRCT is indicated.

While a number of factors (e.g., patient age, occupation, and rehabilitation time) may influence the decision whether a formal rotator cuff repair is required, the major factor considered is the percentage of the tendon thickness torn. This is largely supported clinically by Weber [57] who reported their results in a retrospective study involving patients with PTRCTs involving >50% of the tendon thickness. They demonstrated superior outcomes (higher UCLA score and lower reoperation rate) in patients following rotator cuff repair versus rotator cuff debridement with follow-up time from 2 to 7 years. Subsequent to this, various biomechanical studies have supported this notion. Mazzocca et al. [58] found an increase in rotator cuff strain between intact tendons and articular surface PTRCTs involving >50% of the tendon thickness.

While the percentage of the tendon thickness torn is one factor in determining which operative procedure to perform, other significant variables including age, tear configuration, concomitant pathologies (i.e., labral tear and impingement), and work or sport-related factors (i.e., laborer, sport involved, and position played) may influence the decision to repair a PTRCT. Furthermore, the indications for repair may be different in specific patient populations. Patients experiencing considerable weakness and functional disability may benefit from repair even in tendons with < 50% of the tendon thickness torn, while other patients, such as overhead athletes, may benefit from debridement of tears with 75% of the tendon thickness torn [59].

### 8.1. Arthroscopic Debridement with or without Acromioplasty

Arthroscopic debridement is generally performed in PTRCTs that involve < 50% of the tendon thickness (Grades I and II) and may be combined with or without a concomitant acromioplasty. In the original study by Ellman [60], 50 patients were reviewed following arthroscopic subacromial decompression of which 80% had partial thickness rotator cuff tears. He demonstrated that overall 88% of patients had good to excellent results and suggested that arthroscopic subacromial decompression was a viable option for patients with partial thickness rotator cuff tears. Since that time, several reports have demonstrated good to excellent surgical outcomes, as measured by shoulder specific scales [52, 61–67], with no clear benefit with the addition of a subacromial decompression or acromioplasty [52, 61]. Although these reported results are favorable, the long-term results are unclear. In one study, patients undergoing arthroscopic acromioplasty demonstrated a Constant score 20 points lower than the contralateral shoulder at 101 months after procedure [64].

Similarly, the results of arthroscopic debridement in athletes are variable. In one study of athletes under 40 years of age who were treated with arthroscopic decompression, 86% with acute traumatic injuries had satisfactory outcomes, with an overall 64% return to sport [68]. However, in athletes with an insidious onset of pain, only 45% of patients were able to return to sport. This is further supported in a study by Reynolds et al. [67] who reported that 76% of professional pitchers were able to return to throwing following arthroscopic debridement. However, only 55% were able to return to the same or higher level of play.

Interestingly, some PTRCTs may be more predisposed to failure following arthroscopic debridement and acromioplasty. In 2002, Cordasco et al. [63] reported generally excellent results following arthroscopic subacromial decompression for PTRCTs involving < 50% of the tendon thickness. However, there was a significantly higher failure rate in bursal surface tears (29%) versus articular surface tears (3%). This led the authors to conclude that formal repair may be considered in patients with bursal surface tears involving <50% of the tendon thickness.

While numerous reports have reported favorable results, it does appear that arthroscopic debridement alone or in combination with subacromial decompression does not prevent progression of a PTRCT to a full thickness tear. In a report by Kartus et al. [64], at a mean of 101-month follow-up, 35% of PTRCTs progressed to full thickness tears as evidenced by ultrasound.

Therefore, when performing arthroscopic debridement +/− acromioplasty patients can have improved symptoms with satisfactory clinical outcomes. However, despite these surgical procedures, disease progression can occur. Similar to nonoperative treatment, the indications for arthroscopic debridement +/− acromioplasty again should be weighed against the risk of tear progression.

### 8.2. Arthroscopic Repair

**Figure 2:**
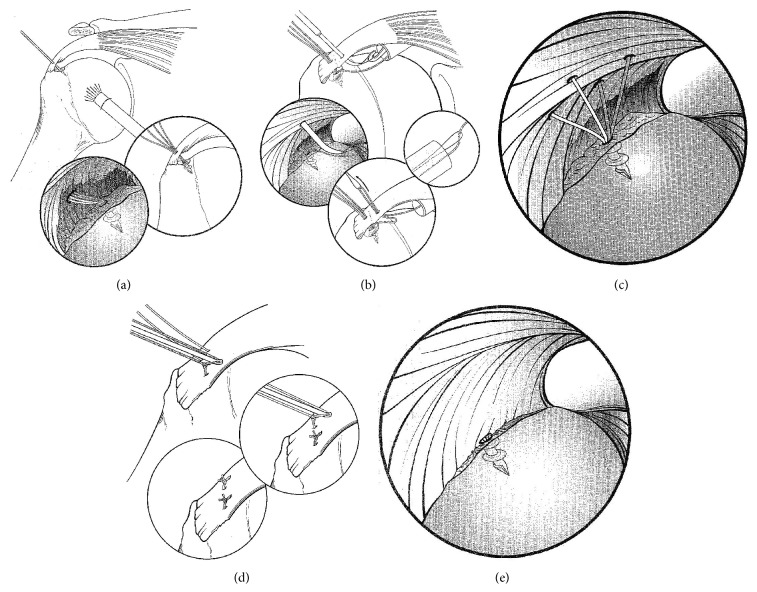
Transtendon repair of a partial thickness articular surface rotator cuff tear. (a) An 18-gauge needle is used to localize the trajectory of the proposed suture anchor. The suture anchor inserted transtendon into the medial aspect of the bone bed. (b) A suture passer is used to shuttle sutures through the intact portion of the rotator cuff. (c) Four suture limbs have been shuttled through the tendon, creating two mattress stitches. (d) The mattress stitches are tied in the subacromial space, reducing the tendon to the bone. (e) After tying all of the sutures, the arthroscope is placed back in the glenohumeral joint to visualize the repair and the reduction of the tendon to the bone (adapted and reprinted from [7] with permission from Elsevier).

Formal arthroscopic rotator cuff repair may be performed utilizing a number of different techniques: conversion repairs [9–14], in situ repairs including transtendon repairs (Figure 2) [18–22], all intra-articular repairs [24], and transosseous repairs [23] (Table 3).

#### 8.2.1. Conversion Repairs

Conversion repair involves completing a PTRCT to a full thickness rotator cuff tear followed by repair. This technique has major advantages of completely removing any devitalized tissue and allowing the utilization of standard rotator cuff repair techniques. This technique has resulted in encouraging outcomes with significant improvement in range of movement, strength, pain relief, and overall function [9–11]. Furthermore, anatomic outcomes utilizing imaging modalities have been favorable. Kamath et al. [11] reported an overall satisfaction rate of 93% following conversion repair with 88% of repairs intact by ultrasound at 11 months (Table 3). Similarly, Iyengar et al. [12] demonstrated significant improvements in UCLA score following conversion repair; 82% of repairs were intact by MRI at 2 years of follow-up. They noted that patients that retore were older, but that a retear did not significantly affect the clinical results. In addition, two more recent conversion repair studies compared the outcomes between bursal versus articular surface partial thickness tears [13, 14]. These studies did not demonstrate a significant difference in retear rates between the two tear locations. Similar to previous reports, both studies demonstrated improved clinical outcomes following conversion repair.

Conversion repair has had successful clinical and anatomic outcomes and has the surgical advantage of using routine rotator cuff repair techniques. However, the theoretical concerns of detaching the residual intact rotator cuff from the greater tuberosity have led surgeons to develop other repair techniques (e.g., in situ repair).

#### 8.2.2. In Situ Repairs

In situ repair techniques have the theoretical advantage of preservation of the existing anatomy by maintaining the intact lateral insertion of the cuff while reestablishing the medial delaminated portion. Although a number of in situ repair techniques have been described, the transtendon repair technique is the most commonly reported technique and is generally performed on articular surface tears (Figure 2). The transtendon technique has demonstrated excellent clinical results with a >90% satisfaction rate (range 91%–98%) (Table 3) [19–22]. Castricini et al. [21] demonstrated that, at a mean follow-up time of 33 months, there were excellent results in 93% of the patients with significant improvements in Constant score. These authors demonstrated optimal tuberosity coverage in all cases with no patients with recurrent tearing on follow-up MRI [21]. Transtendon repair has generally shown good results in athletes, but with a wide range (33% to 89%) of athletes returning to their same level of sport or higher. Patients with poorer results and the inability to return to sport were generally associated with concomitant pathologies such as shoulder instability, SLAP lesions, and bicep tendinopathies [18, 69].

However, it should be noted that, even in patients with excellent outcomes by shoulder specific rating scales, some symptoms might persist. In a study by Castagna et al. [20], the overall patient satisfaction rate was 98% at 2.7 years of follow-up. However, 41% of patients had residual symptoms including complaints of discomfort at the end of range of motion and during daily living activities that required abduction and internal rotation of the shoulder. Patients with residual symptoms were of older age, with an atraumatic onset of symptoms, and had increased tendon retraction with minimal footprint exposure at surgery.

While this residual pain can be multifactorial in nature, some authors have attributed these symptoms to the effect of overtensioning or inappropriate tensioning of the remaining fibers of the rotator cuff to the greater tuberosity (i.e., bursal surface versus articular surface tension mismatch) [70]. This has led to the development of a completely all-inside intra-articular technique, which only reduces the retracted articular fibers to the bone bed and may provide a more anatomic repair [71, 72].

While either in situ technique (i.e., transtendon repair and intra-articular repair) can lead to improved clinical outcomes, the preservation of the intact residual rotator cuff makes surgical repair more demanding and complex. While theoretically advantageous, only a small number of studies have compared conversion repair versus in situ repair techniques.

#### 8.2.3. Conversion versus In Situ Repairs

Various biomechanical studies have evaluated the performance of conversion repairs versus in situ repairs, specifically transtendon repairs. In a cadaveric model of articular surface partial thickness supraspinatus tears, Gonzalez-Lomas et al. [37] demonstrated that, under cyclic loading, gap formation was significantly less and ultimate failure load was significantly higher in the transtendon repair group when compared to the tear conversion with double row rotator cuff repair group. Similarly, in an ovine model of articular surface partial thickness infraspinatus tears, Peters et al. [73] demonstrated that transtendon repair exhibited higher ultimate failure load than double row repair following full thickness conversion.

Although there appears to be a theoretical and biomechanical advantage of transtendon repair over conversion repair, comparative studies have not been able to detect a significant clinical advantage. In a study by Castagna et al. [16], 74 patients were randomized to conversion repair or transtendon repair. Both groups showed significant improvements in Constant score and Visual Analogue Scale with no statistically significant differences between the two groups (Table 3). However, on subgroup analysis, patients who underwent conversion repair had significantly increased postoperative strength scores as compared to patients following transtendon repair. Shin [15] showed that there were similar clinical outcomes between the two repair techniques. Range of motion recovered quicker and patients reported less pain at 3 months comparing conversion repairs to transtendon repairs. While there was some concern with regard to retear rates in patients who underwent conversion repair, a more recent study by Franceschi et al. [17] demonstrated similar retear rates. In a prospective randomized trial, 31 of 32 transtendon repairs and 27 of 28 conversion repairs demonstrated healing on MRI with similar clinical outcomes.

Therefore, while biomechanical studies have suggested a superior mechanical performance, clinically transtendon repair has yet to prove to be more effective than conversion to a full thickness tear with subsequent repair.

## 9. Biologic Adjuncts

In recent years there has been a large interest in the utilization of biologic technologies (e.g., stem cell transplantation, platelet derived growth factor, and platelet rich plasma) in conjunction with the treatment of rotator cuff disease. These therapies generally augment the cellular/matrix proliferation stage of the healing process by increasing or altering the number of growth factors or cells within the healing milieu. While the excitement for such technologies is currently on the rise, there is little clinical evidence to support their use routinely [74]. Furthermore, there are few studies that specifically address the usage of biological adjuncts for PTRCTs.

Cell therapies have been relatively rarely reported but have been utilized in the treatment of rotator cuff disease. In 2013, Wang et al. [75] published a case report of the use of autologous tenocyte implantation in the treatment of an elite athlete with PTRCT which was successfully treated conservatively without repair. In a larger study, Ellera Gomes et al. [76] reported on 14 patients who had bone marrow mononuclear stem cells injected into the tendon margins during repair of full thickness rotator cuff tears. At 12-month follow-up, 14/14 cases demonstrated tendon integrity on MRI with 11/14 sustaining this outcome at 24 months.

The vast majority of literature related to the biological treatment of rotator cuff disease has largely focused on the usage of platelet rich plasma (PRP). While some studies have demonstrated promising results, others have demonstrated no significant difference [74, 77–84]. In a prospective randomized trial, Randelli et al. [78] injected PRP between the tendon-bone interface during rotator cuff repair. Their results demonstrated that, at three months of follow-up, there were initially significantly better pain scores and improved forward elevation in patients treated with PRP. However, by six months there was no significant difference between PRP treated patients and control patients. Similarly, in a prospective randomized trial of 80 patients undergoing rotator cuff repair by Castricini et al. [79], there was no significant difference in Constant score between patients treated with a platelet rich fibrin matrix and controls at a minimum of 16-month follow-up.

Although clinical outcomes do not appear to be significantly different, PRP may improve rotator cuff healing. In a comparative series of 40 patients undergoing rotator cuff repair, Barber et al. [80] demonstrated a significantly lower retear rate in patients treated with a platelet rich fibrin matrix (30% versus 60%). Similarly, in the study by Castricini et al. [79], patients who received a platelet rich fibrin matrix had a lower retear rate (2.5% versus 10.5%), although these values were not statistically significant (*p* = 0.07).

While the above studies have investigated the use of PRP in conjunction with rotator cuff repair, few studies have evaluated its efficacy as a nonoperative treatment modality. In 2013, Kesikburun et al. [82] evaluated the effect of PRP in patients with chronic rotator cuff tendinopathy (i.e., tendinosis or partial thickness rotator cuff tears excluding full thickness rotator cuff tears). In this study, 40 patients were randomized to receive a PRP injection versus saline placebo control. At a one-year follow-up, there was no significant difference in pain, disability, or shoulder range of motion between PRP and saline controls.

Collectively, it appears, at this time, there is little clinical support for the routine use of PRP injections in the treatment of rotator cuff, as both nonoperative and operative treatment modality. This is supported by a recent Cochrane review [81] evaluating the effect of PRP therapy on musculoskeletal injuries. Although the studies were heterogeneous in nature, the authors concluded that the current data failed to show a clinically significant effect on function and pain scores between PRP and control groups.

Furthermore, while PRP is readily available, multiple different methods of extraction, concentration, delivery, and timing protocols limit the generalizability of this technique. This may, in part, be responsible for the mixed results found when utilizing PRP. In the future, further studies are required to determine the clinical effectiveness of PRP or indeed other biological adjuncts prior to their routine use as a treatment modality.

## 10. Conclusion

Partial thickness rotator cuff tears are a common pathology causing disability in a wide range of individuals. There are many treatment options available depending on the size and location of the tear as well as individual patient characteristics. Following failure of nonoperative treatment, the accepted practice is to consider surgical repair in rotator cuff tears that involve 50% or more of the tendon thickness. While a number of surgical techniques have been described, there is insufficient evidence to suggest the use of one repair technique over the other. Furthermore, the use of biologic adjuncts in both nonoperative and operative treatments should be considered investigational.

## Figures and Tables

**Table 1:** Medial to lateral width and anterior to posterior length of the rotator cuff tendon insertions.

Rotator cuff tendon	Medial to lateral width		Anterior to posterior length	
	Mean (mm)	Range (mm)	Mean (mm)	Range (mm)
Supraspinatus	16	12–20	23	18–33
Infraspinatus	18	12–24	28	20–45
Teres minor	21	10–33	29	20–40
Subscapularis	20	15–25	40	35–55

Adapted from [3].

**Table 2:** Classification of partial thickness rotator cuff tears (PTRCTs): articular, bursal, and intratendinous locations.

Grade	Size of tear (percentage of tendon thickness)
I	<3 mm (<25%)
II	3–6 mm (25–50%)
III	>6 mm (>50%)

Adapted from [8].

**Table 3:** Clinical and Anatomic Outcomes of Arthroscopic Repair of Partial Thickness Rotator Cuff Tears (PTRCTs).

Study	Number of patients	Type of repair	Clinical Outcome	Clinical Outcome	Anatomic Outcome
			Preoperative → Postoperative follow-up score (measure)	Percentage of patients satisfied	Percentage of repairs intact (imaging method)
Porat et al. (2008) [9]	36	Conversion	17.24 → 31.47 (UCLA)		

Deutsch (2007) [10]	41	Conversion	42 → 93 (ASES)	98%	

Kamath et al. (2009) [11]	42	Conversion	46.1 → 82.1 (ASES)	93%	88% intact (ultrasound)

Iyengar et al. (2011) [12]	22	Conversion	19.1 → 32.9 (UCLA)		82% intact (MRI)

Kim et al. (2013) [13]	54	Bursal surface Conversion	6.7 → 1.4 (VAS) 14.7 → 30.9 (UCLA) 36.1 → 90.7 (ASES) 4.7 → 10.0 (SST)		89% intact (MRI)
	29	Articular surface Conversion	5.8 → 0.9 (VAS) 15.7 → 30.5 (UCLA) 42.4 → 90.4 (ASES) 5.1 → 9.7 (SST)		92% intact (MRI)

Kim et al. (2014) [14]	21	Bursal surface Conversion	5.38 → 1.19 (VAS) 19.81 → 32.52 (UCLA) 47.78 → 90.80 (ASES) 57.38 → 83.00 (Constant)		90.5% intact (MRI)
	20	Articular surface Conversion	4.95 → 1.05 (VAS) 19.80 → 32.70 (UCLA) 48.69 → 91.80 (ASES) 51.00 → 75.85 (Constant)		100% intact (MRI)

Shin (2012) [15]	24	Conversion	5.3 → 1.1 (VAS) 49.2 → 86.2 (ASES) 59.0 → 87.1 (Constant)	92%	92% intact (MRI)
	24	Transtendon	5.5 → 1.4 (VAS) 50.8 → 89.1 (ASES) 54.8 → 84.8 (Constant)	92%	100% intact (MRI)

Castagna et al. (2015) [16]	37	Conversion	3.6 (ΔVAS) 29 (Δ Constant)		
	37	Transtendon	3.4 (ΔVAS) 25.1 (Δ Constant)		

Franceschi et al. (2013) [17]	28	Conversion	47 → 90 (ASES) 46 → 91 (Constant)		96% intact (MRI)
	32	Transtendon	46 → 91 (ASES) 48 → 92 (Constant)		97% intact (MRI)

Ide et al. (2005) [18]	17	Transtendon	17.3 → 32.9 (UCLA)		

Waibl and Buess (2005) [19]	22	Transtendon	17.1 → 31.2 (UCLA)	91%	

Castagna et al. (2009) [20]	54	Transtendon	14.1 → 32.9 (UCLA) 9.8 → 0.8 (SST) 45.3 → 90.6 (Constant)	98%	

Castricini et al. (2009) [21]	31	Transtendon	44.4 → 91.6 (Constant)	93%	100% intact (MRI)

Seo et al. (2011) [22]	24	Transtendon Double-row	6.6 → 0.6 (VAS) 38 → 89 (ASES)	92%	

Tauber et al. (2008) [23]	16	Transosseous	15.8 → 32.8 (UCLA)	94%	

Spencer et al. (2010) [24]	20	Intra-articular	74 → 92 (PSS)		

VAS = Visual Analog Scale.

UCLA = University of California at Los Angeles.

ASES = American Shoulder and Elbow Surgeons.

SST = Simple Shoulder Test.

PSS = Penn Shoulder Score.
